# Methionine Alters the Fecal Microbiota and Enhances the Antioxidant Capacity of Lactating Donkeys

**DOI:** 10.3390/ani15050648

**Published:** 2025-02-23

**Authors:** Fei Huang, Zongjie Ma, Xinyi Du, Changfa Wang, Guiqin Liu, Miaomiao Zhou

**Affiliations:** College of Agriculture and Biology, Liaocheng Research Institute of Donkey High-Efficiency Breeding and Ecological Feeding, Liaocheng University, Liaocheng 252000, China; huangfei080616@163.com (F.H.); 18764659740@163.com (Z.M.); duxinyi1289@163.com (X.D.); wangchangfa@lcu.edu.cn (C.W.); guiqinliu@lcu.edu.cn (G.L.)

**Keywords:** lactating donkeys, methionine, antioxidant capacity, microbiota, correlation analysis

## Abstract

This study looked at how methionine (Met) affects on serum biochemical indicators, antioxidant indicators, and the fecal microbiota of lactating donkeys. A total of 18 healthy donkeys at the mid-lactation stage were used in this study. They were given concentrate meals with varying concentrations of Met after being randomly split into three groups: Met group I (M1) received 5 g/d of Met, Met group II (M2) received 15 g/d of Met, and the control group (C) received 0 g/d of Met. The trial lasted for five weeks in total. Fecal microbes, antioxidant markers, and blood biochemical indicators were examined. The findings showed that while the levels of malondialdehyde (MDA) decreased (*p* < 0.05), the addition of Met (15 g/d) significantly enhanced the total antioxidant capacity (T-AOC) and catalase (CAT) activity. Milk samples from the different groups trended towards having significant differences in the levels of the serum biochemical indicators albumin (ALB) and lactate dehydrogenase (LDH) (0.05 < *p* < 0.1). A fecal microbiome analysis revealed that the addition of Met (5 g/d) increased the abundance of *Methanocorpusculum* and *Ruminococcus*, and the addition of 15 g/d of Met increased the abundance of *Ruminococcus*, *Peptococcus*, and *Anaeroplasma* while decreasing the abundance of the *[Eubacterium]_ruminantium_group*. A correlation analysis revealed that *Ruminococcus* and *Methanocorpusculum* were positively correlated with the T-AOC and CAT activity (M1 group). *Peptococcus* was significantly negatively correlated with MDA levels and positively correlated with the T-AOC (M2 group). *Anaeroplasma* was positively correlated with CAT activity (M2 group). The above results indicated that Met increased donkey antioxidant levels and modulated the abundance of the fecal microbial community. These findings lay the groundwork for boosting the production performance and general health of nursing donkeys.

## Simple Summary

Donkeys are specialized economic animals, and their meat, milk, and skin are of great nutritional and commercial value. Although methionine (Met) is an essential amino acid for nursing animals, its effects on the antioxidant capacity and rectal microbial abundance of lactating donkeys have not been studied. Adding Met to the diet could enhance the lactation performance of lactating donkeys. In this study, we examined how dietary Met affected nursing donkeys’ fecal bacteria makeup and blood biochemical markers. The findings demonstrated that feeding donkeys Met raised their antioxidant levels and controlled the number of fecal microorganisms. This study offers a foundation for enhancing the performance and general health of nursing donkeys.

## 1. Introduction

With the development of society and changes in social demands, the primary use for donkeys has gradually shifted from being used for labor to providing meat [1] and dairy products [2]. Donkey meat is fresh and tasty, and because of its high protein and low fat and cholesterol contents, donkey meat is more suitable than beef and pork for making salami and fermented meat products [3,4]. In one study, the nutritional value of donkey milk and milk products was shown to be very high [5]. Zhou et al. (2023) reported that donkey milk contains several beneficial bacteria, including Proteobacteria and Ralstonia, and many bioactive proteins that have antioxidant activity and antimicrobial properties [6,7]. Kallis et al. (2018) reported that human milk and donkey milk have similar chemical compositions. Donkey milk is well tolerated by infants (82.6–88%), and can be easily adapted for infants with an allergy to cow milk proteins [8]. Milk production in donkeys, however, has been reported to be approximately 1–1.5 kg/d [9]. The low production of donkey milk limits its commercial application. Additionally, oxidative stress is common in nursing donkeys, leading to a decline in milk production [10]. Excessive lipolysis and the synthesis of huge amounts of milk (increased milk production) during breastfeeding cause metabolic levels to rise noticeably [11]. An increase in metabolic levels increases the risk of oxidative stress (development of udder inflammation) and limits the lactation capacity [12]. Reducing oxidative stress and enhancing the health and performance of nursing donkeys may be achieved by appropriate dietary management during lactation.

Amino acids are recognized to be crucial for controlling oxidative and immunological conditions [13]. According to He et al. (2018), the primary functions of amino acids in intestinal inflammation are to enhance the intestinal barrier, reduce intestinal damage, prevent oxidative stress, and suppress the production of pro-inflammatory cytokines [14]. Zhou et al. (2021) also performed preliminary studies on donkey oligopeptide transport [15]. The role of dietary methionine (Met) supplementation in maintaining the antioxidant status [16], immune status [17], and milk production performance [18] of dairy cows has been widely studied. Interestingly, dietary Met supplementation also increased milk production and milk component yield in lactating donkeys [19]. Therefore, Met can function as a nutritional substrate and regulatory factor. There are many studies on the use of Met as a nutritional factor in lactating animals; however, there are no studies on the effects of Met supplementation on the health status of lactating donkeys. It is therefore not clear whether the addition of Met would have a positive effect on the overall health status. This study set out to investigate how adding Met to the diet affects antioxidant levels and flora regulation in lactating donkeys and to analyze the mechanisms of their interactions.

## 2. Materials and Methods

### 2.1. Ethical Approval of Animal Protocols

The Liaocheng University Scientific Research Ethic Committee examined and approved the animal use methodology (20211002).

### 2.2. Experimental Procedure and Treatments

The experimental procedure of Huang et al. (2024) [19] was used. In this study, 18 healthy Dezhou donkeys (273.4 ± 30 kg) from Liaocheng City, Shandong Province, China, were employed; the females had already given birth to two or three foals. Every donkey was kept in its own enclosure (semi-enclosed). The trial lasted five weeks in total, including a one-week pre-feeding interval. The 18 lactating donkeys were divided into three groups of six each: the M1 group was given 5 g/d of Met, the control group (C) was given 0 g/d of Met (99%, Digoxa, Kissingen, Germany), and the M2 group was given 15 g/d of Met. Every nursing donkey received enough water and the same amount of grass hay—nearly 6 kg—as well as 2.5 kg of concentrate each day. The nutritional values and concentrate composition are shown in Table 1. Dry matter was the foundation of the concentrate feed’s nutritional makeup.

### 2.3. Sample Collection and Storage

At the end of the experimental period, blood and uncontaminated feces in the rectum were collected. On the morning of the final day of the experiment following a 12 h fast, blood and feces were taken, and the feces were frozen in liquid nitrogen. Using an anticoagulant tube, blood samples were extracted from the female donkeys’ jugular veins. The top serum layer was then extracted by centrifuging the samples for 10 min at 3500 rpm/min. After that, the serum and feces samples were frozen in liquid nitrogen and kept at −80 °C until they were analyzed.

### 2.4. 16S rRNA Gene Sequencing of the Fecal Microbiota

In this experiment, using certain primers (515F: 5′-CCTAYGGGRBGCASCAG-3′; 806R: 5′-GGACTACNNGGTATCTAAT-3′) and barcodes, the 16S rRNA gene in the V3-V4 region was amplified [20]. The PCR reactions were performed using a T100 gradient PCR instrument (Bio-Rad, Hercules, CA, USA). The PCR reaction mixture consisted of Phusion High-Fidelity PCR Master Mix (15 µL), DNA (10 ng), and primers (0.2 µM). The following were the conditions for cycling: 1 min at 98 °C, followed by 30 cycles at 98 °C (10 s), 50 °C (30 s), 72 °C (30 s), and finally 5 min at 72 °C. The PCR products were mixed and then extracted using the Gel Extraction Kit (QIAGEN, New York, NY, USA).

The PCR products were processed using a kit. An Illumina Novaseq sequencing device (Illumina, San Diego, CA, USA) was used to perform double-end sequencing on the small fragment libraries that were created based on the features of the amplified areas. The obtained valid data were then subjected to species annotation and abundance analysis, which revealed the species composition of the samples; additional alpha and beta diversity analyses, which can be used to explore the differences in community structure between samples; read splicing and filtering; operational taxonomic unit clustering; or amplicon sequence variant (ASV) noise reduction. Individual analysis and in-depth data mining were then conducted in accordance with the project needs. Phylogenetic trees were created, the sample data were homogenized, multiple sequences were compared with ASVs, and differences in community structure across samples or groups were investigated using dimensionality reduction techniques such as sample clustering tree construction and PCA. To further explore the differences in community structure among the grouped samples, statistical methods such as *t*-tests and LEfSe analysis were chosen to assess the significance of the species composition and community structure changes.

### 2.5. Serum Biochemical Indicators and Antioxidant Indicators

The methods for serum preparation and determination of blood biochemical and antioxidant indicators of Zhang et al. (2023) [21] were used. The concentrations of serum biochemical parameters were analyzed using an Automatic Biochemistry Analyzer (Indiko, Thermo Scientific, Waltham, MA, USA). Total protein (TP), albumin (ALB), triglyceride (TG), urea, blood glucose (GLU), aspartate amino transferase (AST), glutamic pyruvic transaminase (ALT), and lactate dehydrogenase (LDH) levels were the blood biochemical markers that were measured. Colorimetric assay kits were used to measure the levels of glutathione peroxidase (GSH-Px), superoxide dismutase (SOD), catalase (CAT), the total antioxidant capacity (T-AOC), and malondialdehyde (MDA) in the serum.

### 2.6. Bioinformatics Analysis

The bioinformatics analysis method of Zhu et al. (2021) [22] was used. The raw 16s rRNA gene sequencing data were quality-filtered using FASTQ (v0.20.0). FLASH (v1.2.7) was used to combine the sequencing reads. Sequences were processed and clustered using USEARCH (v7.0) in order to analyze the operational taxonomic units (OTUs). The OTUs obtained by clustering the 16S rRNA gene rDNA sequences were employed as estimates of the microbiological taxa. QIIME (v1.9.1) was used to select each OTU’s most prevalent sequence. The representative sequence of each OTU with 97% similarity and a taxonomy with a minimum threshold of 0.7 was designated using the RDP Classifier (v2.2).

### 2.7. Statistics Analysis

Mothur (v1.30.1) was used to perform the alpha diversity analysis, which included the Shannon and Chao indices to assess the evenness and richness of the OTUs, respectively. To show how the two groups in this study differed in terms of variety at various levels, the Mann–Whitney test was used. The cut-off point for rejecting the null hypothesis was set at *p* < 0.05. Each group’s OTUs at various levels were evaluated using a community bar graph. To account for the variation and separation between groups according to the Bray–Curtis techniques, QIIME (v1.9.1) was used to perform a principal coordinate analysis (PCoA), a component of beta diversity. Linear discriminant analysis (LDA) effect size (LEfSe) using an LDA threshold score of >3 was used to determined which taxa and OTUs were more connected to each group and contributed to the differences.

The data were analyzed using SPSS 25.0 software and Graphpad Prism 9. The statistical significance of the group differences was assessed using the Student’s *t* test procedure. The mean and standard error of the mean (SEM) were used to express the test findings. *p* < 0.05 indicates a significant difference, 0.05 < *p* < 0.10 indicates a significant trend, and *p* > 0.10 indicates no meaningful difference.

## 3. Results

### 3.1. Effects of Met on Serum Biochemical Parameters

**Figure 1:**
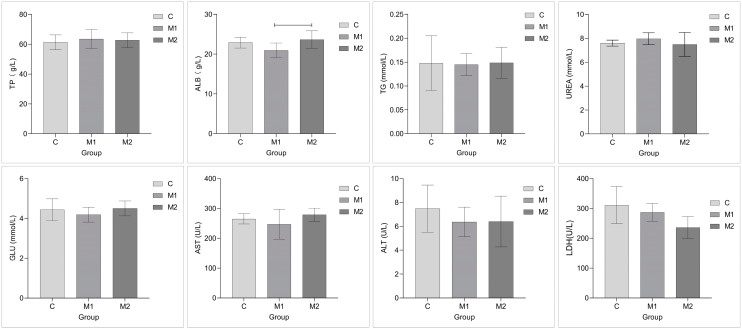
The effects of dietary Met supplementation on female donkeys’ blood biochemical indicators. TP: total blood protein; ALB: albumin; TG: triglyceride; UREA: blood urea nitrogen; GLU: blood glucose; AST: aspartate transaminase; ALT: alanine transaminase; LDH: lactate dehydrogenase. Number of test animals: 6 per group. The method used for the statistical analysis of the experimental data was ANOVA. 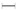: 0.05 < *p* < 0.10 between groups.

Figure 1 illustrates how the addition of Met altered the biochemical characteristics of the donkeys’ blood. The addition had an impact on the levels of ALB and LDH. The ALB level exhibited a clear decreasing trend in the M1 group compared to that of the M2 group (0.05 < *p* < 0.10); the ALB levels in the C group compared to that of the M1 group (*p* = 0.25) and in the C group compared to that of the M2 group (*p* = 0.78) were not significantly different. Compared with that in the C group, the concentration of LDH in the M2 group showed a significant decreasing trend (0.05 < *p* < 0.10); the concentration of LDH in C compared to that of the M1 group (*p* = 0.68) and in the M1 group compared to that of the M2 group (*p* = 0.22) were not significantly different. The other blood biochemical indicators, however, did not show significant changes (*p* > 0.10).

### 3.2. Serum Antioxidant Capacity

**Figure 2:**
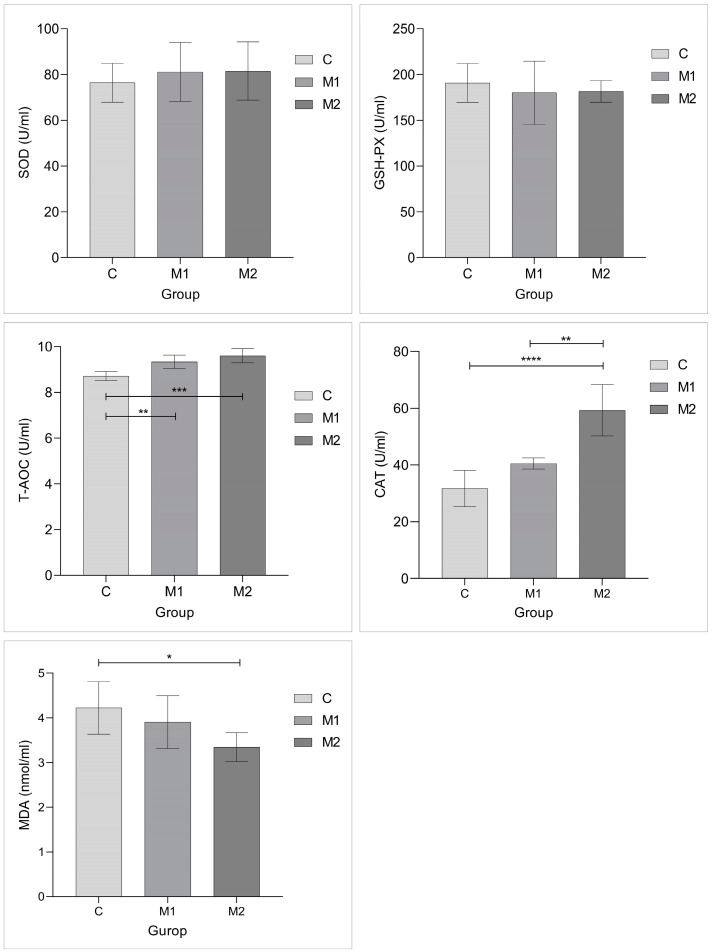
The effects of dietary Met supplementation on antioxidant indices in female donkeys. The column height indicates the concentration of the corresponding antioxidant indicator. CAT: catalase; MDA: malondialdehyde; SOD: superoxide dismutase; GSH-PX: glutathione peroxidase; and T-AOC: total antioxidant capacity. Number of test animals: 6 per group. The method used for the statistical analysis of the experimental data was ANOVA. * *p* < 0.05, ** *p* < 0.01, *** *p* < 0.001, **** *p* < 0.0001 between groups.

The changes in blood antioxidant indicators in lactating donkeys are displayed in Figure 2. The concentrations of T-AOC, CAT, and MDA changed significantly with Met supplementation (*p* < 0.05). The addition of Met considerably raised the serum T-AOC concentration in comparison to that of the C group (*p* < 0.01). The addition of Met significantly increased the activity of serum CAT (*p* < 0.01), with the highest activity observed in the M2 group (*p* < 0.01). The addition of Met (15 g/d) resulted in a significant decrease in the MDA concentration (*p* < 0.05). Furthermore, there was no difference in the SOD and GSH-PX levels across the three groups (*p* > 0.10).

### 3.3. Fecal Microbiological Analysis

#### 3.3.1. Microbiome Overview

**Figure 3:**
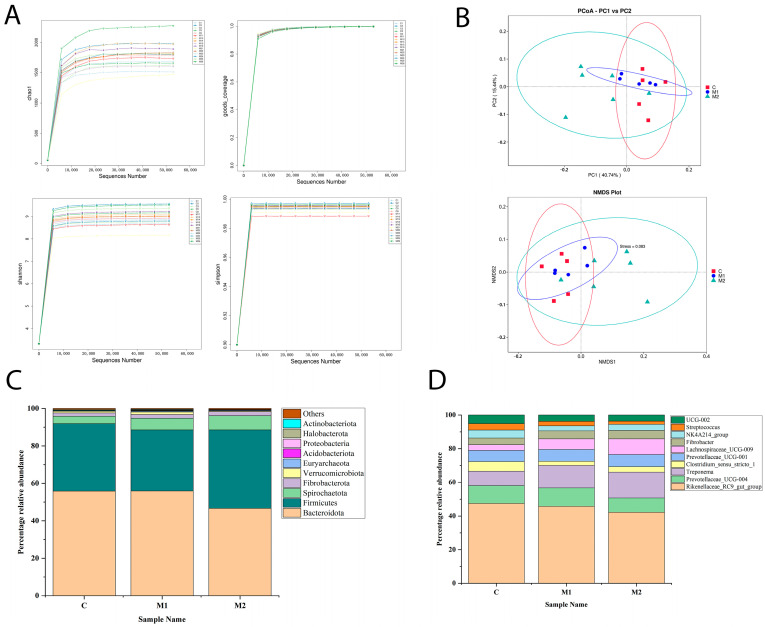
The effects of Met supplementation on the microbial diversity and abundance of fecal microorganisms in female donkeys: (**A**) alpha diversity according to the Shannon, Simpson, Chao 1, and Goods Coverage indices and (**B**) beta diversity according to the PCoA and NMDS results. Each point in the NMDS plot indicates a sample. The distance between points indicates the size of the difference. Samples in the same group are indicated by the same color. A stress value less than 0.2 indicates that the NMDS results could accurately reflect the size of the difference among the samples. (**C**) The relative abundance of the microbes at the phylum level. (**D**) The relative abundance of the microbes at the genus level.

A satisfactory sequencing depth for the fecal microbiota content analysis was shown by the fact that over 98.0% of the valid data had a data quality score > Q20 after the invalid 16S rDNA sequences of all samples were filtered out. According to the alpha diversity dilution curves (Figure 3A), the data values for every sample were within the typical range. Among the frequently employed techniques for beta diversity analysis are PCoA and NMDS. The PCoA revealed no significant difference in species composition across the three groups in this experiment (Figure 3B) (*p* > 0.05), and the NMDS mapping analysis also confirmed the species composition similarity (*p* < 0.05).

The microorganisms at the phylum and genus levels were used to assess the composition of the fecal microbiota. The microbiota found in this study were present in more than half of the biological replicates in each group and had a relative abundance higher than 0.1%. The results at the phylum level revealed that *Bacteroidetes* and *Firmicutes* dominated the groups (Figure 3C). The results of the genus-level analysis revealed that the dominant groups were the *Rikenellaceae_RC9_gut_group*, *Prevotellaceae_UCG-004*, *Treponema, Prevotellaceae_UCG-001*, and *Lachnospiraceae_UCG-009* (Figure 3D).

#### 3.3.2. Microbial Differential Analysis

**Figure 4:**
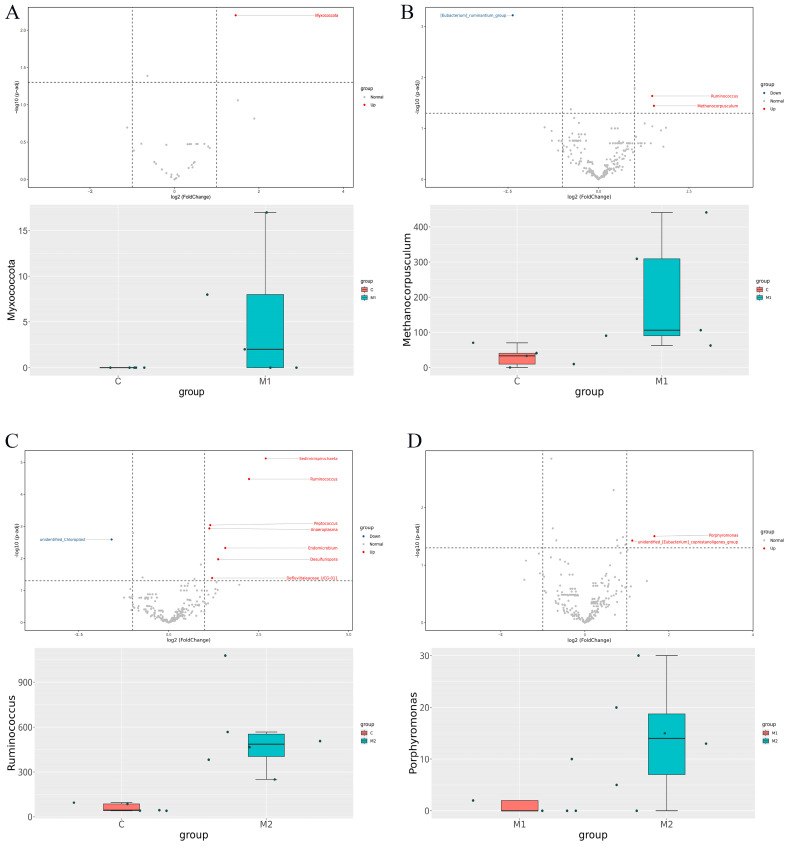
The effects of Met supplementation on the microbial variability in the feces of female donkeys. (**A**) The significant differences at the phylum level in the M1 group vs. the C group are shown in the volcano and box plots. (**B**) The significant differences at the genus level in the M1 group vs. the C group are shown in the volcano and box plots. (**C**) The significant differences at the genus level in the M2 group vs. the C group are shown in the volcano and box plots. (**D**) The significant differences at the genus level in the M2 group vs. the M1 group are shown in the volcano and box plots.

The results at the phylum level revealed a significant increase in the relative abundance of Myxococcota in the M1 group (Figure 4A, *p* < 0.01). The genus-level analysis revealed that the relative abundances of *Methanocorpusculum* (Figure 4B) in the M1 group and *Ruminococcus* (Figure 4C) in the M2 group were significantly greater (*p* < 0.05) than those in Group C. Furthermore, compared to the M1 group, the abundance of *Porphyromonas* was higher (*p* < 0.05) in the M2 group (Figure 4D). The differential microorganisms are shown in Table 2.

### 3.4. Correlation Analysis Between the Fecal Microbiome and Blood Indicators

**Figure 5:**
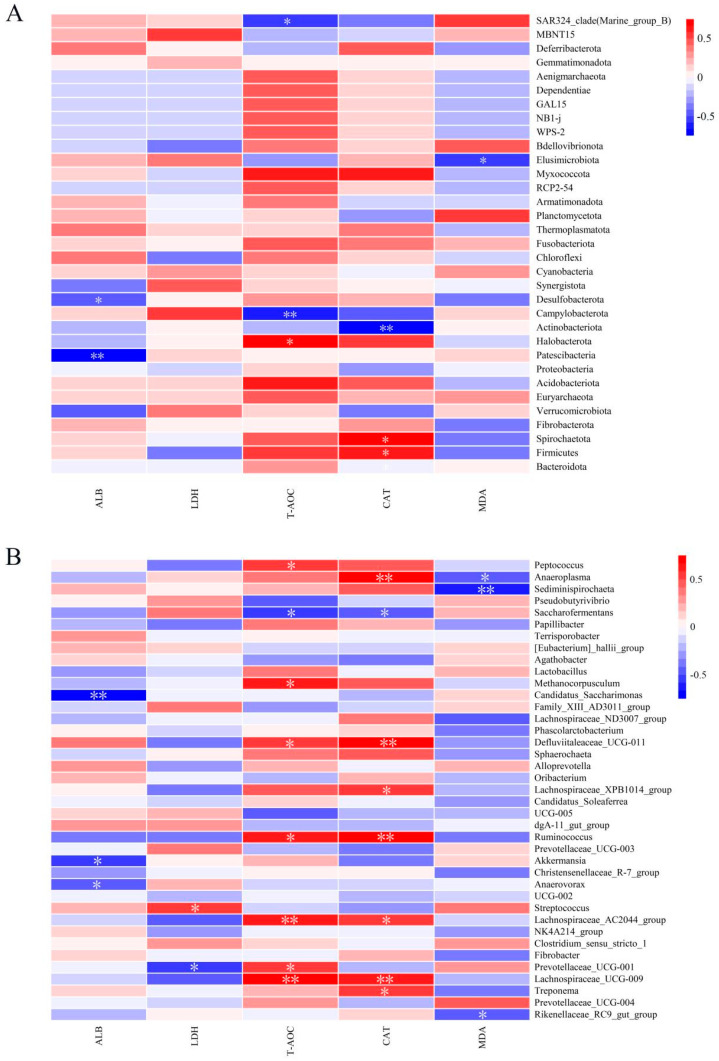
Spearman’s correlations between fecal microbiota and serum indicators. (**A**) Heatmap of phylum correlations. (**B**) Heatmap of genus correlations. Blue denotes a negative correlation, whereas red denotes a positive correlation. The Spearman correlation’s strength is indicated by the color intensity. ** *p* < 0.01 and * *p* < 0.05.

The correlations between the fecal microbiome and blood indicators were analyzed using the Spearman algorithm (Figure 5). According to the phylum-level heatmap (Figure 5A), the T-AOC was negatively correlated with Campylobacterota (*p* < 0.01). The T-AOC was significantly positively correlated the genera *Ruminococcus*, *Defluviitaleaceae_UCG-011*, and *Methanocorpusculum* (Figure 5B, *p* < 0.05). Furthermore, CAT activity was significantly positively correlated with the *Ruminococcus*, *Defluviitaleaceae_UCG-011*, and *Anaeroplasma* abundances (*p* < 0.05). The MDA level was negatively correlated with the *Rikenellaceae_RC9_gut_group*, *Sediminispirochaeta*, and *Anaeroplasma* (*p* < 0.05). *Prevotellaceae_UCG-001* and LDH levels had a negative correlation (*p* < 0.05), whereas *Anaerovorax*, *Akkermansia*, and *Candidatus_Saccharimonas* had a negative correlation (*p* < 0.05) with ALB levels.

## 4. Discussion

### 4.1. Effects of Met Supplementation on Serum Parameters

An increase or decrease in the blood biochemical indicators, including serum urea, TP, AST, ALT, and total cholesterol levels, can reflect the health status of a donkey, especially for the routine indicators ALB and LDH [23]. An increase in ALB levels represents an improvement in the nutritional status of the organism [24]. However, an increase in LDH levels is usually indicative of the presence of a relevant pathology in the organism [25]. Liu et al. (2022) reported that the dietary addition of bile acids to piglets increased ALB levels, which promoted their intestinal development [26]. Interestingly, Luo et al. (2023) reported that the addition of ultra-ground Astragalus membranaceus could have a modulatory effect on the immune system in weaned goats by increasing the ALB concentration [27]. Zhuang et al. (2020) reported that the addition of selenomethionine to bovine mammary epithelial cell cultures could reduce *Escherichia coli*-induced inflammation by decreasing LDH levels and triggering the selenoprotein S-mediated TLR4/NF-kB signaling cascade [28]. In this study, the addition of Met to the diet resulted in an increasing trend in ALB levels (0.05 < *p* < 0.1) and a decreasing trend in LDH levels (0.05 < *p* < 0.1) in the blood of lactating donkeys. Our results are consistent with the above findings. These results indicate that the addition of the Met can improve the health status (inflammation, immunity, and gut health statuses) of lactating donkeys.

Furthermore, an increase in the serum antioxidant capacity (as measured by the T-AOC, CAT activity, and MDA levels) can prevent oxidative stress in lactating animals and improve their lactation performance [10,29]. Additionally, the results of Huang et al. (2024) revealed that an increase in the serum antioxidant capacity was an important factor for improving donkey lactation performance [19]. According to the current study, feeding Met to nursing donkeys raised their T-AOC and CAT activity while lowering their MDA levels, which is in line with the results obtained in dairy cows supplemented with rumen-protecting Met [30]. These findings suggested that feeding lactating donkeys Met can raise their blood antioxidant levels, which is beneficial for animal health and improving their lactating performance.

### 4.2. Effects of Met Supplementation on Fecal Microorganisms

Studying and analyzing the gut microbiota of lactating animals can help improve animal health and productivity [31]. Additionally, the microbiological composition affects milk yield and milk composition in lactating animals [32]. This study’s findings showed that feeding Met to nursing donkeys considerably raised the levels of the genera *Methanocorpusculum*, *Ruminococcus*, and *Peptococcus* while decreasing the abundance of *Eubacterium ruminantium*. *Methanocorpusculum*, a methanogenic bacterium, was found to be associated with organismal antioxidant enzymes in studies on anaerobic digestion of lignocellulosic biomass [33]. Interestingly, *Methanocorpusculum* was also found to be positively correlated with antimicrobial and antioxidant effects and negatively correlated with the *Eubacterium ruminantium* group in a study on the intestinal health of laying hens [34]. The increased abundance of *Ruminococcus* implied improved milk production performance and antioxidant capacity in dairy goats [35]. In a study by Ma et al. (2022), the abundance of *Ruminococcus* in the intestinal tract of sows was positively correlated with oxidative stress and the inflammatory response [36]. *Peptococcus* is positively correlated with antioxidant capacity [37]. The results of Francesco et al. (2021) also revealed that the abundance of *Peptococcus* was positively correlated with body weight [38]. Interestingly, Kong et al. (2022) reported that *Peptococcus* was associated with reduced inflammation and an increased antioxidant capacity in broilers [39]. Similarly, the *[Eubacterium]_ruminantium_group* was associated with lamb growth performance, inflammation, and apoptosis in nutritional experiments in lambs [40]. This study’s findings demonstrated that adding Met to donkey meals improved the health of nursing donkeys by controlling the presence of certain fecal bacteria linked to antioxidant activity.

### 4.3. Intermodulation of Microbiological and Biochemical Indices in Donkeys

Guo et al. (2024) reported that serum antioxidant indices (T-AOC and CAT activity) were correlated with microbial abundance [41]. The same results were also found in this study. In the present study, the serum antioxidant activity of lactating donkeys was significantly increased by the addition of Met, and the microbiome results revealed a change in the abundance of the bacterial flora. The correlation analysis of the two indices revealed that *Ruminococcus* was positively and significantly correlated with the T-AOC and CAT activity. *Methanocorpusculum* was positively correlated with the T-AOC. The abundance of *Peptococcus* was negatively correlated with the MDA content but positively correlated with the T-AOC. *Anaeroplasma* was positively and significantly correlated with CAT activity (*p* < 0.01). Many studies have reached the same conclusion. It was found that *Ruminococcus* was positively correlated with antioxidant indices (T-AOC and CAT activity) in studies on yellow-feathered broilers [42,43], dairy goats [35], Holstein dairy cows [44], and suckling piglets [36]. Ma et al. (2022) reported that *Methanocorpusculum* was positively correlated with antioxidant properties [34]. Similarly, Zhang et al. reported that *Anaeroplasma* was positively correlated with antioxidant indicators [45]. In studies on pigs, *Peptococcus* was found to be negatively correlated with the antioxidant indicator MDA and positively correlated with the T-AOC [37,46]. A combination of correlation and microbiome analyses revealed that the bacterial flora was positively correlated with antioxidant indices. In conclusion, the addition of Met was able to increase the abundance of *Ruminococcus*, *Methanocorpusculum*, *Peptococcus*, and *Anaeroplasma* flora, thereby increasing the antioxidant indices such as the T-AOC and CAT activity, and thus the antioxidant capacity of lactating donkeys.

## 5. Conclusions

The addition of Met significantly increased the abundance of fecal *Ruminococcus*, *Methanocorpusculum*, *Peptococcus*, and *Anaeroplasma* in lactating donkeys. Also, the Met supplementation improved the antioxidant capacity of lactating donkeys by increasing their antioxidant indices (T-AOC, CAT activity, etc.). The abundances of *Ruminococcus*, *Methanocorpusculum*, *Peptococcus*, and *Anaeroplasma* were positively correlated with the antioxidant capacity. This is the first study on the effects of Met supplementation on the physical performance of lactating donkeys, which lays the foundation for improving the health status of lactating donkeys and improving donkey milk production performance.

## Figures and Tables

**Table 1:** The nutritional levels and concentrate composition of the experimental meals.

Materials, %	
Sorghum	40.60
Soybean meal	27.00
Wheat bran	21.00
Stone flour	6.30
Calcium hydrogen phosphate	1.60
Salt	0.70
Sodium bicarbonate	0.50
Vitamin complex	0.10
Mineral complex	1.00
Choline chloride	0.30
Lysine	0.90
**Chemical Composition, %**	
Dry matter	87.80
Crude protein	18.20
Crude fiber	3.73
Crude ash	12.02
Crude fat	2.79
Calcium	0.74
Total phosphorus	0.74
Non-phytate phosphorus	0.43
Na	0.76
Lysine	1.38

Note: Met (added) or Met in the ingredient list is the weight of additional Met added as a percentage of the total concentrate feed.

**Table 2:** Microorganism differences between the M2, M1, and C groups.

Classification	Groups	Differential Microorganism	LogFC	*p*-Value	Up/Down
Phylum	M1 vs. C	Myxococcota	1.45	0.0063	up
	M1 vs. C	Methanocorpusculum	1.53	0.0361	up
Genus		Ruminococcus	1.48	0.0231	up
		[Eubacterium]_ruminantium_group	−2.37	0.0006	down
	M2 vs. C	Sediminispirochaeta	2.69	0.0000	up
		Ruminococcus	2.23	0.0000	up
		Endomicrobium	1.57	0.0047	up
		Desulfurispora	1.38	0.0106	up
		Defluviitaleaceae_UCG−011	1.21	0.0409	up
		Peptococcus	1.16	0.0009	up
		Anaeroplasma	1.13	0.0011	up
		unidentified_Chloroplast	−1.58	0.0026	down
	M2 vs. M1	Porphyromonas	1.66	0.0313	up
		unidentified_[Eubacterium]_coprostanoligenes_group	1.13	0.0375	down

Note: logFC: logarithmic value (base 2) of the fold change between the groups.

## Data Availability

The data were not deposited in a public repository. The data are available upon reasonable request directly from the corresponding authors.
